# [μ_2_-Bis(di­phenyl­phosphan­yl)hexa­ne]bis­[undeca­carbonyl-*triangulo*-triruthenium(3 *Ru*—*Ru*)] hexane monosolvate: crystal structure and Hirshfeld surface analysis

**DOI:** 10.1107/S2056989017014517

**Published:** 2017-10-20

**Authors:** Omar bin Shawkataly, Siti Syaida Sirat, Mukesh M. Jotani, Edward R. T. Tiekink

**Affiliations:** a Chemical Sciences Programme, School of Distance Education, Universiti Sains Malaysia, 11800 USM, Penang, Malaysia; b Department of Physics, Bhavan’s Sheth R. A. College of Science, Ahmedabad, Gujarat 380001, India; c Research Centre for Crystalline Materials, School of Science and Technology, Sunway University, 47500 Bandar Sunway, Selangor Darul Ehsan, Malaysia

**Keywords:** crystal structure, ruthenium, cluster, carbon­yl, Hirshfeld surface analysis

## Abstract

In the title cluster complex hexane solvate, [Ru_6_(C_30_H_32_P_2_)(CO)_22_]·C_6_H_14_, two Ru_3_(CO)_11_ fragments are linked by a Ph_2_P(CH_2_)_6_PPh_2_ bridge with the P atoms equatorially disposed with respect to the Ru_3_ triangle in each case; the hexane solvent mol­ecule is statistically disordered. The Ru⋯Ru distances span a relatively narrow range, *i.e*. 2.8378 (4) to 2.8644 (4) Å. The hexyl chain within the bridge has an all-*trans* conformation. In the mol­ecular packing, C—H⋯O inter­actions between cluster mol­ecules, and between cluster and hexane solvent mol­ecules lead to a three-dimensional architecture. In addition, there are a large number of C≡O⋯π(arene) inter­actions in the crystal. The importance of the carbonyl groups in establishing the packing is emphasized by the contribution of 53.4% to the Hirshfeld surface by O⋯H/H⋯O contacts.

## Abstract 2

The title crystal features two Ru_3_(CO)_11_ fragments linked by a Ph_2_P(CH_2_)_6_PPh_2_ bridge, the latter with an all-*trans* conformation. The mol­ecular packing features C—H⋯O, as well as C≡O⋯π(arene) inter­actions.

## Chemical context   

In the realm of cluster chemistry, diphosphane ligands are known to maintain the integrity of the metal core during chemical reactions (Kabir & Hogarth, 2009 ▸). In the solid state, diphosphane ligands are known to adopt a variety of bonding modes towards triruthenium clusters, including monodentate, chelating, edge-bridging and linking two clusters (Bruce *et al.*, 1982 ▸; Lozano Diz *et al.*, 2001 ▸; Shawkataly *et al.*, 2012 ▸). The motivation for studying triruthenium cluster complexes containing diphosphane ligands arises primarily due to these complexes making attractive starting materials for further reactivity studies (Kabir & Hogarth, 2009 ▸; Rajbangshi *et al.*, 2015 ▸, Shawkataly *et al.*, 2016 ▸). Despite this, only relatively few compounds with diphosphane ligands connecting two tri­ruthenium clusters have been structurally characterized (Bruce *et al.*, 1982 ▸; Van Calcar *et al.*, 1998 ▸; O’Connor *et al.*, 2003 ▸; Kakizawa *et al.*, 2015 ▸). Our inter­est in synthesizing the title [Ru_3_(CO)_11_]_2_[Ph_2_P(CH_2_)_6_PPh_2_] cluster is to enable a com­parison of the structural variations that arise from lengthening of the organic backbone in the diphosphane ligand. Furthermore, the joining of smaller cluster units with such spacer ligands is a useful method for the construction of larger aggregates (Bruce *et al.*, 1985 ▸; Kakizawa *et al.*, 2015 ▸). In the present study, two triruthenium cluster units were successfully connected through a bidentate bridging Ph_2_P(CH_2_)_6_PPh_2_ ligand in the compound [Ru_3_(CO)_11_]_2_[Ph_2_P(CH_2_)_6_PPh_2_], which was isolated as a 1:1 *n*-hexane solvate, (I). Herein, the crystal and mol­ecular structures of (I) are described, as well as an analysis of the calculated Hirshfeld surface.
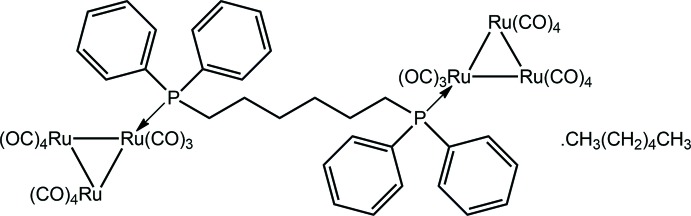


## Structural commentary   

**Figure 1:**
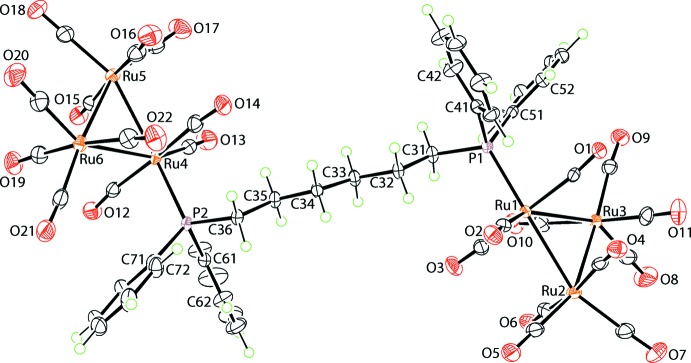
The mol­ecular structure of the Ru_6_ cluster mol­ecule in (I), showing the atom-labelling scheme and displacement ellipsoids at the 50% probability level.

The mol­ecular structure of the cluster mol­ecule in (I) is shown in Fig. 1 ▸. The asymmetric unit comprises two Ru_3_(CO)_11_ cluster mol­ecules linked by a Ph_2_P(CH_2_)_6_PPh_2_ bridge and a hexane mol­ecule which is statistically disordered over two sets of sites. The phosphane P atom occupies a position effectively coplanar with the Ru_3_ core in each case, *i.e*. an equatorial site. The two Ru_3_ cluster residues are each constructed about a triangular Ru_3_ core, and the Ru–Ru edges span a relatively narrow range of distances, *i.e*. 2.8378 (4) Å for Ru2⋯Ru3 to 2.8644 (4) Å, for Ru1⋯Ru3. Each of the carbonyl ligands occupies a terminal position, with the Ru—C≡O angles ranging from 169.7 (4)° for Ru—C10≡O10 to 179.4 (4)° for Ru5—C18≡O18. The hexyl chain in the diphosphane ligand has an all-*trans* conformation, with the P1/P2—C—C—C torsion angles being −177.8 (3) and 175.5 (2)°, respectively, and the C—C—C—C torsion angles ranging from 173.7 (3)° for C33—C34—C35—C36 to −177.4 (3)° for C32—C33—C34—C35. The consequence of this is that the pairs of P-bound phenyl rings lie to either side of the chain.

## Supra­molecular features   

**Figure 2:**
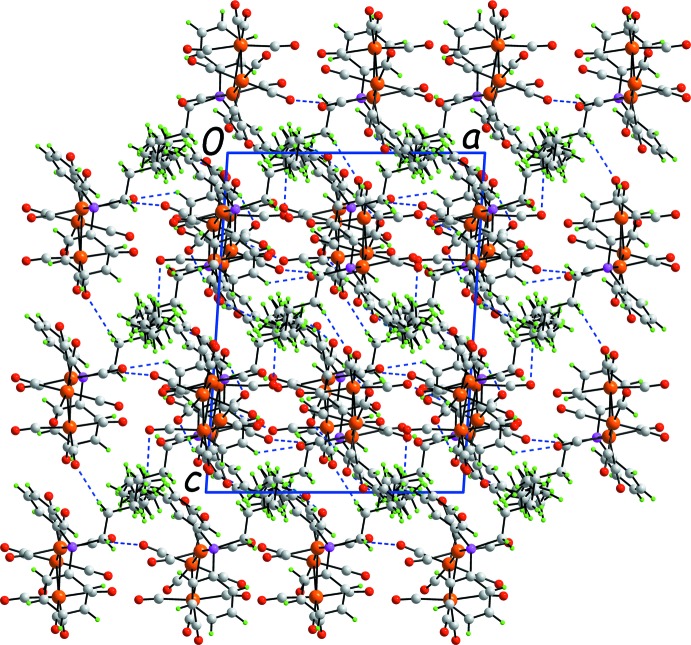
A view of the unit-cell contents shown in projection down the *b* axis. The C—H⋯O inter­actions are shown as blue dashed lines.

The mol­ecular packing of (I) comprises a complex network of C—H⋯O and C≡O⋯π inter­actions. The C—H donors for the C—H⋯O inter­actions are either methyl­ene- or phenyl-H, Table 1 ▸, and by themselves define a three-dimensional architecture, Fig. 2 ▸. Additional stability to the crystal is provided by a number of C≡O⋯π(arene) inter­actions, either with end-on or side-on approaches. Further discussion and details of the identified C≡O⋯π(arene) inter­actions are found below in *Analysis of the Hirshfeld surface* (§4). The closest inter­actions between the cluster mol­ecule and the solvent hexane mol­ecule are of the type solvent-methyl­ene-C—H⋯O(carbon­yl), Table 1 ▸. The solvent mol­ecules reside in cavities defined by the cluster mol­ecules.

## Analysis of the Hirshfeld surface   

**Figure 3:**
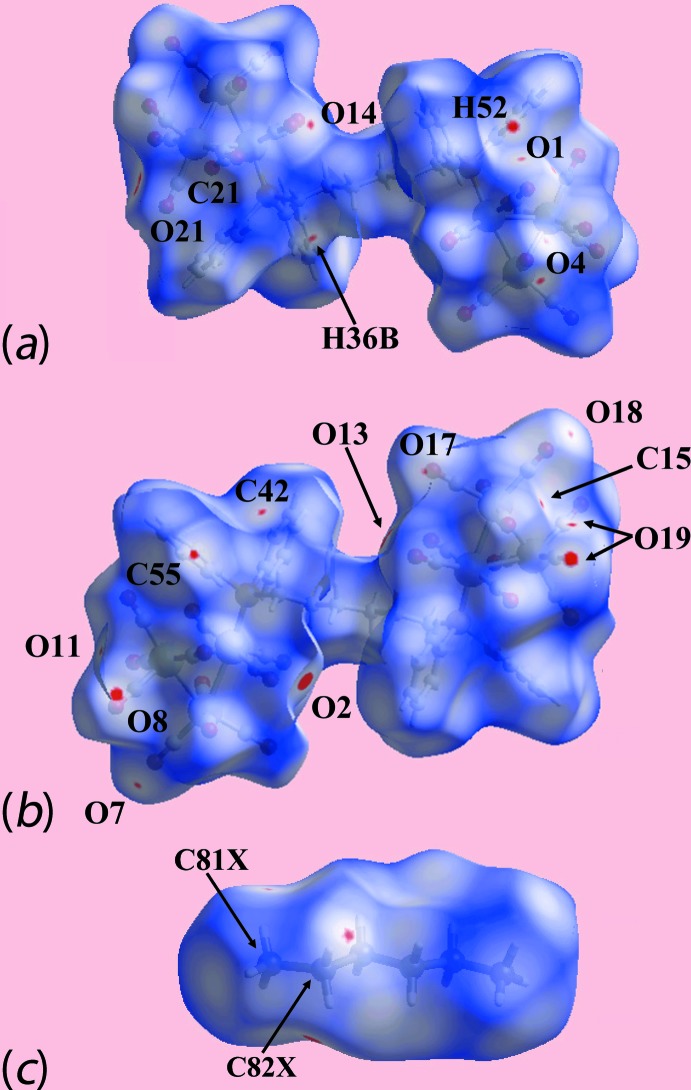
Views of the Hirshfeld surface mapped over *d*_norm_: (*a*) and (*b*) showing different orientations of the Ru_6_ cluster mol­ecule in (I) over the range −0.062 to 1.417 au, and (*c*) for the solvent hexane mol­ecule in the range −0.033 to 1.345 au.

**Figure 4:**
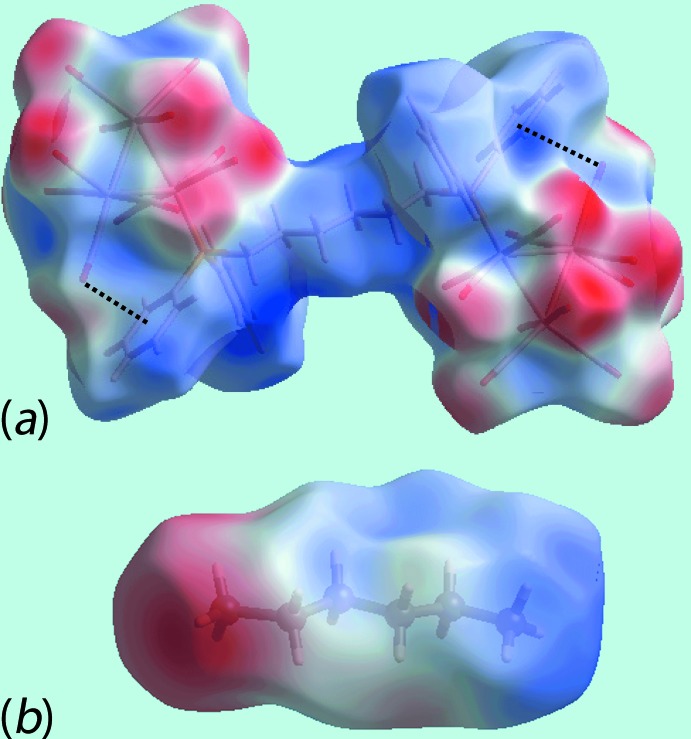
Views of the Hirshfeld surface mapped over the electrostatic potential for (*a*) the Ru_6_ cluster mol­ecule in (I), in the range ±0.046 au, and (*b*) the solvent hexane mol­ecule in the range ±0.147 au. The red and blue regions represent negative and positive electrostatic potentials, respectively.

**Figure 5:**
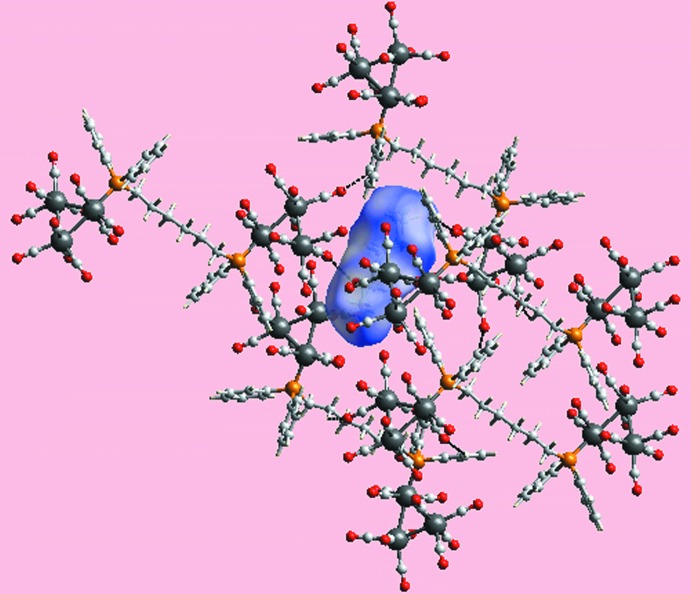
A view of Hirshfeld surface mapped over *d*_norm_ about a hexane mol­ecule within a cavity defined by Ru_6_-cluster mol­ecules and showing inter­molecular C—H⋯O contacts as black dashed lines.

The Hirshfeld surface calculations of (I) were performed in accord with a recent publication on a related heavy-atom complex and its dioxane solvate (Jotani *et al.*, 2017 ▸). The presence of the carbonyl groups in (I) lead to their participation in C—H⋯O, C≡O⋯π and C⋯O/O⋯C inter­actions, and the Hirshfeld surfaces mapped over *d*_norm_, Fig. 3 ▸, indicate the influence of these in the crystal. Of the C—H⋯O inter­actions summarized in Table 1 ▸, the donors and acceptors of more influential contacts are viewed as bright-red spots near the phenyl-H52 and C55, diphosphane-hexyl-H32*B* and C82*X*, and carbonyl-O4, O8, O14 and O19 atoms, whereas the comparatively weak C—H⋯O contacts are viewed as faint-red spots near the phenyl-C42, hexane-C81*X* and C82*X*, and carbonyl-O7, O11 and O17 atoms in Fig. 3 ▸. In addition, the presence of bright-red spots near the O2, O13, O21 and C21 atoms and the diminutive-red spots near the O1, O4, O19 and C15 atoms in Fig. 3 ▸, are also indicative of short inter-atomic O⋯O and C⋯O/O⋯C contacts effective in the crystal. The donors and acceptors of inter­molecular inter­actions can also be viewed as blue and red regions, respectively, on the Hirshfeld surface mapped over electrostatic potential for the cluster mol­ecule in Fig. 4 ▸ *a*, and for the hexane mol­ecule in Fig. 4 ▸ *b*. Two intra­molecular C—O⋯π contacts, *i.e*. one between carbonyl-O9 and the phenyl C51–C56 ring, and the other between carbonyl-O21 and the phenyl C71–C76 ring are also illustrated through black, dotted lines in Fig. 4 ▸ *a*. The cavity occupied by the hexane mol­ecule, showing the relevant C—H⋯O contacts, Table 1 ▸, is highlighted in Fig. 5 ▸.

**Figure 6:**
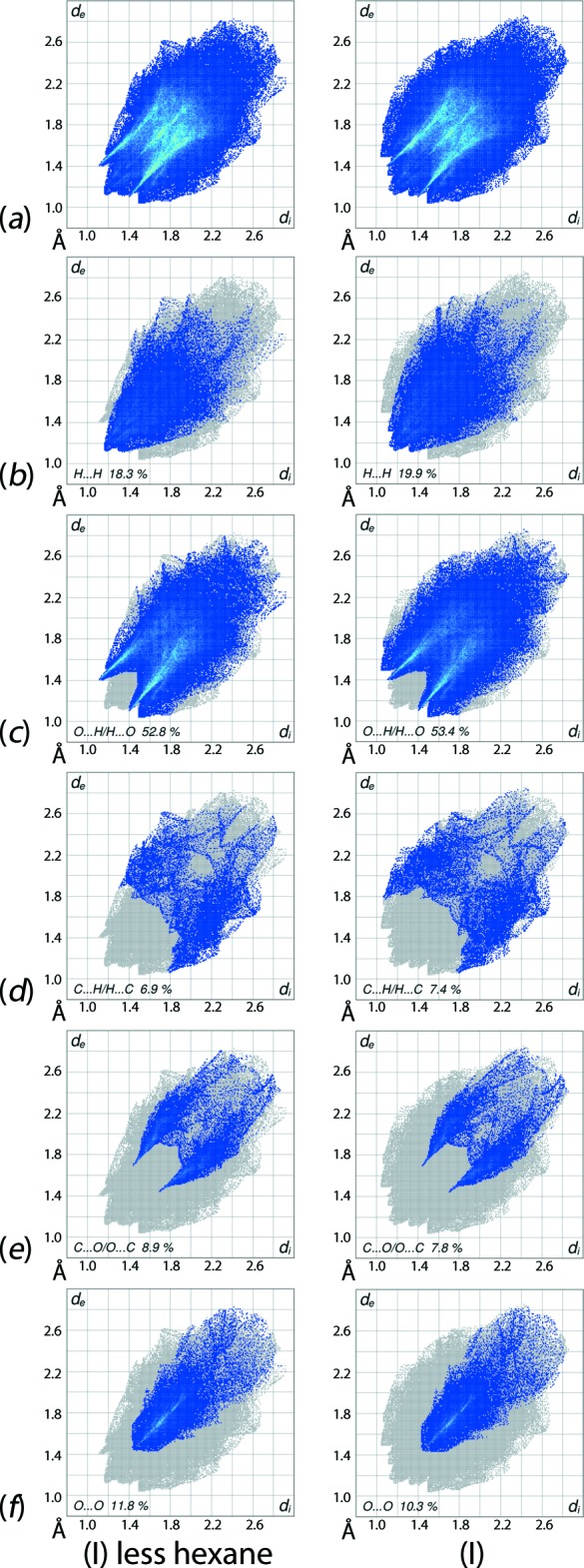
(*a*) The full two-dimensional fingerprint plots for (I) less hexane (left-hand column) and for (I), and those delineated into (*b*) H⋯H, (*c*) O⋯H/H⋯O, (*d*) C⋯H/H⋯C, (*e*) C⋯O/O⋯C and (*f*) O⋯O contacts.

The overall two-dimensional fingerprint plots for the cluster mol­ecule alone and for (I) are shown in Fig. 6 ▸ *a* and clearly indicate the significance of the solvent mol­ecule on the packing. This is also evident from the percentage contribution from the different surface contacts summarized in Table 2 ▸ and from the fingerprint plots delineated into H⋯H, O⋯H/H⋯O, C⋯H/H⋯C, C⋯O/O⋯C and O⋯O contacts (McKinnon *et al.*, 2007 ▸) in Figs. 6 ▸ *b*–*f*, respectively. The inclusion of the hexane mol­ecule in the Hirshfeld surface calculations increases the relative contributions from O⋯H/H⋯O, H⋯H and C⋯H/H⋯C contacts but decreases those contributed by O⋯O and C⋯O/O⋯C contacts. This observations arises as a result of the participation of the solvent mol­ecule in inter­atomic H⋯H and C⋯H/H⋯C contacts, Table 3 ▸, and in the inter­molecular C—H⋯O inter­actions listed in Table 1 ▸.

The two pairs of short peaks at *d*_e_ + *d*_i_ ∼ 2.3 and 2.4 Å in the fingerprint plot for (I) delineated into H⋯H contacts, Fig. 6 ▸ *b* (right-column) indicate the presence of short inter­atomic contacts involving phenyl- and hexane-hydrogen atoms. The greatest contribution of 53.4% to the Hirshfeld surface of (I) is from O⋯H/H⋯O contacts and these are characterized as two specific types of inter­actions leading to two distinct distributions of points in the delineated fingerprint plot of Fig. 6 ▸ *c*. The pair of sharp spikes having green aligned points within the plot and with tips at *d*_e_ + *d*_i_ ∼ 2.5 Å are the result of C—H⋯O inter­actions involving cluster-bound atoms as donors and acceptors; the points corresponding to short inter­atomic weak C—H⋯O contacts (Table 1 ▸) and O⋯H/H⋯O contacts (Table 3 ▸) are merged within the plot. On the other hand, the exterior portion with broad tips at *d*_e_ + *d*_i_ ∼ 2.6 Å are due to C—H⋯O inter­actions involving hexane-bound atoms as donors and carbonyl- oxygen atoms as acceptors. The comparison of O⋯H/H⋯O delineated fingerprint plots for in Fig. 6 ▸ *c* confirm this observation.

The involvement of hexane-H83*A* and H83*B* atoms in the short inter­atomic C⋯H/H⋯C contacts (Table 3 ▸) results in forceps-like peaks at *d*_e_ + *d*_i_ ∼ 2.9 Å in the delineated fingerprint, Fig. 6 ▸ *d*. The 7.8% contribution from C⋯O/O⋯C contacts to the Hirshfeld surface of (I) is due to the involvement of all carbonyl-O atoms (except O5) either in short inter­atomic C⋯O/O⋯C contacts, Table 3 ▸, or in end-on or side-on C≡O⋯π inter­actions, summarized in Table 4 ▸. The impact of end-on metal-C≡O⋯π(arene) inter­actions upon supra­molecular aggregation patterns has been addressed in the recent literature (Zukerman-Schpector *et al.*, 2011 ▸, 2012 ▸). The pair of sharp, forceps-like tips at *d*_e_ + *d*_i_ ∼ 3.0 Å in the fingerprint plots delineated into C⋯O/O⋯C contacts, Fig. 6 ▸ *e*, represent short C⋯O/O⋯C contacts involving carbonyl-O1, O13, C15 and C21 atoms while the points distributed in adjoining parabolic form around (*d*_e_, *d*_i_) = (1.8, 2.0 Å) and (2.0, 1.8 Å) represent C≡O⋯π inter­actions, Table 4 ▸. The fingerprint plot delineated into O⋯O contacts, Fig. 6 ▸ *f*, has a distribution of points within the rocket-shape with the tip at *d*_e_ + *d*_i_ ∼ 2.9 Å, extending up to 3.0 Å, and is the result of significant short O⋯O contacts summarized in Table 3 ▸. The small contribution from C⋯C contacts on the Hirshfeld surfaces of (I) has a negligible effect on the packing.

## Database survey   

The most closely related structure in the literature is that of the dppe (Ph_2_PCH_2_CH_2_PPh_2_) analogue, *i.e*. Ru_3_(CO)_11_(dppe)Ru_3_(CO)_11_ (Van Calcar *et al.*, 1998 ▸). The centrosymmetric mol­ecule presents the same key features as described above for the cluster mol­ecule in (I). There are only a handful of structures whereby two triangular clusters are bridged by a Ph_2_P(CH_2_)_6_PPh_2_ ligand as in (I). The most closely related of these to the present report is formulated as Fe_3_(CO)_11_(Ph_2_P(CH_2_)_6_PPh_2_)Fe_3_(CO)_11_ (Ferguson *et al.*, 1991 ▸). The difference in this centrosymmetric mol­ecule, *cf*. (I), is that there are two μ_2_-bridging carbonyls connecting the Fe atom bonded to P to one of the other Fe atoms of the triangle; the remaining Fe atom is bound to four terminal carbonyl ligands as in (I).

## Synthesis and crystallization   

The reagents Ru_3_(CO)_12_ (200.0 mg, 0.0003 mol) and Ph_2_P(CH_2_)_6_PPh_2_ (70.0 mg, 0.0002 mol) were mixed in distilled tetra­hydro­furan (25 ml). The reaction mixture was treated dropwise with sodium di­phenyl­ketyl solution until the colour of the mixture turned from orange to dark-red followed by stirring for 30 min. The reaction was monitored by thin-layer chromatography (TLC). The solvent was removed under reduced pressure and the product was separated by preparative TLC (2:3 di­chloro­methane:*n*-hexa­ne) to afford three bands. The second band was characterized as [Ru_3_(CO)_11_]_2_(Ph_2_P(CH_2_)_6_PPh_2_). Orange laths were grown by solvent/solvent diffusion of CH_2_Cl_2_/*n*-hexane at 283 K. Analysis calculated for C_52_H_32_O_22_P_2_Ru_6_·C_6_H_14_: C 39.51, H 2.63%; found: C 38.45, H 1.53%. ATR–IR [cm^−1^]: ν(CO) 2093 (*s*), 2038 (*m*), 1957 (*br*). ^1^H NMR (CDCl_3_): δ 7.52–7.41 (*m*, 20H, Ph), 2.37–2.33 (*m*, 4H, CH_2_), 1.26–1.13 (*m*, 8H, CH_2_). ^31^P{^1^H} (CDCl_3_): δ 26.83 (*s*).

## Refinement   

Crystal data, data collection and structure refinement details are summarized in Table 5 ▸. The carbon-bound H atoms were placed in calculated positions (C—H = 0.95–0.99 Å) and were included in the refinement in the riding model approximation, with *U*_iso_(H) set to 1.2–1.5*U*_eq_(C). The hexane mol­ecule was statistically disordered over two sites and the atomic positions of each were refined independently but, the C—C bond lengths for each component were refined with the distance restraint C—C = 1.50±0.005 Å. The anisotropic displacement parameters were restrained to be almost isotropic and those for matching atoms to be similar. Owing to poor agreement, one reflection, *i.e*. 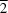
54, was omitted from the final cycles of refinement. The maximum and minimum residual electron density peaks of 2.43 and 1.32 e Å^−3^, respectively, were located 1.34 and 0.50 Å from the C22 and Ru6 atoms, respectively.

## Supplementary Material

Crystal structure: contains datablock(s) I, global. DOI: 10.1107/S2056989017014517/hb7713sup1.cif

Structure factors: contains datablock(s) I. DOI: 10.1107/S2056989017014517/hb7713Isup2.hkl

CCDC reference: 975445

Additional supporting information:  crystallographic information; 3D view; checkCIF report

## Figures and Tables

**Table 1:** Hydrogen-bond geometry (Å, °) *Cg*1 and *Cg*2 the ring centroids of the C41–C46 and C51–C56 rings, respectively.

*D*—H⋯*A*	*D*—H	H⋯*A*	*D*⋯*A*	*D*—H⋯*A*
C35—H35*B*⋯O20^i^	0.99	2.60	3.297 (5)	128
C36—H36*B*⋯O4^ii^	0.99	2.54	3.393 (4)	144
C42—H42⋯O7^iii^	0.95	2.56	3.401 (5)	148
C52—H52⋯O19^iv^	0.95	2.52	3.448 (4)	167
C55—H55⋯O14^i^	0.95	2.54	3.278 (5)	134
C62—H62⋯O18^v^	0.95	2.59	3.501 (5)	161
C82*X*—H82*D*⋯O8^vi^	0.99	2.55	3.391 (16)	143
C81*X*—H81*F*⋯O17^vii^	0.98	2.59	3.48 (2)	150
C82*X*—H82*C*⋯O11^viii^	0.99	2.59	3.528 (14)	157

Symmetry codes: (i) 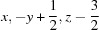; (ii) 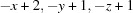; (iii) 
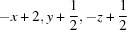
; (iv) 
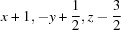
; (v) 
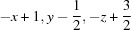
; (vi) 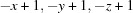; (vii) 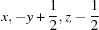; (viii) 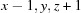.

**Table 2:** Percentage contributions of interatomic contacts to the Hirshfeld surfaces for (I) without hexane and for (I)

Contact	(I) without hexa­ne	(I)
O⋯H/H⋯O	52.8	53.4
H⋯H	18.3	19.9
O⋯O	11.8	10.3
C⋯O/O⋯C	8.9	7.8
C⋯H/H⋯C	6.9	7.4
C⋯C	1.3	1.2

**Table 4:** Summary of short interatomic C≡O⋯π contacts (Å, °) in (I) *Cg*1–*Cg*4 the ring centroids of the C41–C46, C51–C56, C61–C66 and C71–C76 rings, respectively.

C	O	*Cg*	O⋯*Cg*	C—O⋯*Cg*	C⋯*Cg*	Symmetry operation
C3	O3	*Cg*3	3.756 (4)	161.0 (3)	4.839 (5)	1 − *x*, 1 − *y*, 1 − *z*
C7	O7	*Cg*2	3.564 (4)	100.1 (3)	3.928 (4)	2 − *x*, − 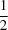 + *y*, 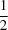 − *z*
C9	O9	*Cg*2	3.228 (3)	94.2 (2)	3.499 (4)	*x*, *y*, *z*
C18	O18	*Cg*4	3.707 (3)	97.6 (3)	4.015 (5)	1 − *x*, 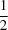 + *y*, 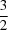 − *z*
C19	O19	*Cg*1	3.554 (3)	146.0 (3)	4.546 (5)	−1 + *x*, 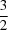 − *y*, 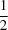 + *z*
C20	O20	*Cg*3	3.671 (4)	146.6 (4)	4.664 (4)	*x*, 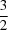 − *y*, 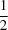 + *z*
C21	O21	*Cg*4	3.074 (4)	98.3 (3)	3.424 (4)	*x*, *y*, *z*

**Table 3:** Summary of short inter-atomic (Å) in (I)

Contact	Distance	Symmetry operation
O1⋯C15	3.196 (5)	1 + *x*, 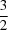 − *y*, − 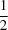 + *z*
O1⋯O19	3.009 (4)	1 + *x*, 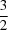 − *y*, − 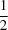 + *z*
O2⋯O2	2.900 (4)	2 − *x*, 1 − *y*, 1 − *z*
O4⋯O18	3.024 (4)	1 + *x*, 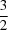 − *y*, − 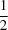 + *z*
O7⋯H42	2.62	2 − *x*, − 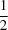 + *y*, 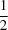 − *z*
O8⋯H43	2.62	2 − *x*, − 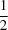 + *y*, 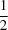 − *z*
O13⋯O21	2.970 (5)	*x*, 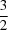 − *y*, − 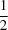 + *z*
O13⋯C21	3.156 (5)	*x*, 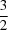 − *y*, − 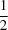 + *z*
C8⋯H83*B*	2.86	1 + *x*, *y*, −1 + *z*
C10⋯H83*A*	2.87	1 − *x*, 1 − *y*, 1 − *z*
H35*B*⋯H63	2.38	1 − *x*, 1 − *y*, 1 − *z*
H55⋯H86*D*	2.39	1 − *x*, 1 − *y*, 1 − *z*
H73⋯H84*C*	2.30	1 − *x*, 1 − *y*, 2 − *z*

**Table 5:** Experimental details

Crystal data	
Chemical formula	[Ru_6_(C_30_H_32_P_2_)(CO)_22_]·C_6_H_14_
*M*_r_	1763.31
Crystal system, space group	Monoclinic, *P*2_1_/*c*
Temperature (K)	100
*a*, *b*, *c* (Å)	14.5323 (4), 23.3731 (6), 19.1883 (4)
β (°)	93.653 (1)
*V* (Å^3^)	6504.3 (3)
*Z*	4
Radiation type	Mo *K*α
μ (mm^−1^)	1.48
Crystal size (mm)	0.61 × 0.48 × 0.09

Data collection	
Diffractometer	Bruker SMART APEXII CCD area-detector
Absorption correction	Multi-scan (*SADABS*; Bruker, 2009 ▸)
*T*_min_, *T*_max_	0.466, 0.880
No. of measured, independent and observed [*I* > 2σ(*I*)] reflections	78116, 19875, 14916
*R*_int_	0.047
(sin θ/λ)_max_ (Å^−1^)	0.716

Refinement	
*R*[*F* ^2^ > 2σ(*F* ^2^)], *wR*(*F* ^2^), *S*	0.043, 0.097, 1.01
No. of reflections	19875
No. of parameters	851
No. of restraints	232
H-atom treatment	H-atom parameters constrained
Δρ_max_, Δρ_min_ (e Å^−3^)	2.43, −1.32

Computer programs: *APEX2* and *SAINT* (Bruker, 2009 ▸), *SHELXS97* (Sheldrick, 2008 ▸), *SHELXL2014* (Sheldrick, 2015 ▸), *ORTEP-3 for Windows* (Farrugia, 2012 ▸), *DIAMOND* (Brandenburg, 2006 ▸) and *publCIF* (Westrip, 2010 ▸).

## supplementary crystallographic information

### Crystal data

**Table:**

[Ru_6_(C_30_H_32_P_2_)(CO)_22_]·C_6_H_14_	*F*(000) = 3456
*M**_r_* = 1763.31	*D*_x_ = 1.801 Mg m^−^^3^
Monoclinic, *P*2_1_/*c*	Mo *K*α radiation, λ = 0.71073 Å
*a* = 14.5323 (4) Å	Cell parameters from 9961 reflections
*b* = 23.3731 (6) Å	θ = 2.3–30.5°
*c* = 19.1883 (4) Å	µ = 1.48 mm^−^^1^
β = 93.653 (1)°	*T* = 100 K
*V* = 6504.3 (3) Å^3^	Lath, orange
*Z* = 4	0.61 × 0.48 × 0.09 mm

### Data collection

**Table:**

Bruker SMART APEXII CCD area-detector diffractometer	19875 independent reflections
Radiation source: fine-focus sealed tube	14916 reflections with *I* > 2σ(*I*)
Graphite monochromator	*R*_int_ = 0.047
φ and ω scans	θ_max_ = 30.6°, θ_min_ = 1.4°
Absorption correction: multi-scan (*SADABS*; Bruker, 2009)	*h* = −20→20
*T*_min_ = 0.466, *T*_max_ = 0.880	*k* = −33→24
78116 measured reflections	*l* = −27→27

### Refinement

**Table:**

Refinement on *F*^2^	232 restraints
Least-squares matrix: full	Hydrogen site location: inferred from neighbouring sites
*R*[*F*^2^ > 2σ(*F*^2^)] = 0.043	H-atom parameters constrained
*wR*(*F*^2^) = 0.097	*w* = 1/[σ^2^(*F*_o_^2^) + (0.0336*P*)^2^ + 15.8643*P*] where *P* = (*F*_o_^2^ + 2*F*_c_^2^)/3
*S* = 1.01	(Δ/σ)_max_ = 0.003
19875 reflections	Δρ_max_ = 2.43 e Å^−^^3^
851 parameters	Δρ_min_ = −1.32 e Å^−^^3^

### Special details

**Table:**

Geometry. All esds (except the esd in the dihedral angle between two l.s. planes) are estimated using the full covariance matrix. The cell esds are taken into account individually in the estimation of esds in distances, angles and torsion angles; correlations between esds in cell parameters are only used when they are defined by crystal symmetry. An approximate (isotropic) treatment of cell esds is used for estimating esds involving l.s. planes.

### Fractional atomic coordinates and isotropic or equivalent isotropic displacement parameters (Å^2^)

**Table:**

	*x*	*y*	*z*	*U*_iso_*/*U*_eq_	Occ. (<1)
Ru1	1.00620 (2)	0.55309 (2)	0.32612 (2)	0.01502 (6)	
Ru2	1.03733 (2)	0.43753 (2)	0.28780 (2)	0.01907 (6)	
Ru3	1.02396 (2)	0.52319 (2)	0.18283 (2)	0.01861 (6)	
Ru4	0.44772 (2)	0.73619 (2)	0.66392 (2)	0.01666 (6)	
Ru5	0.43523 (2)	0.85302 (2)	0.70390 (2)	0.02018 (7)	
Ru6	0.45413 (2)	0.76646 (2)	0.80837 (2)	0.02177 (7)	
P1	0.95575 (6)	0.64831 (4)	0.33370 (4)	0.01513 (17)	
P2	0.49391 (6)	0.64017 (4)	0.66137 (4)	0.01546 (17)	
O1	1.19315 (19)	0.60410 (11)	0.29484 (15)	0.0285 (6)	
O2	1.0800 (2)	0.52828 (12)	0.47441 (13)	0.0290 (6)	
O3	0.8119 (2)	0.50828 (13)	0.35164 (16)	0.0366 (7)	
O4	1.22972 (19)	0.47647 (12)	0.34408 (15)	0.0292 (6)	
O5	0.9991 (2)	0.38167 (12)	0.42725 (15)	0.0364 (7)	
O6	0.8389 (2)	0.41183 (14)	0.22927 (17)	0.0411 (8)	
O7	1.1266 (3)	0.33838 (13)	0.21263 (18)	0.0459 (9)	
O8	0.9924 (3)	0.43736 (14)	0.06391 (17)	0.0514 (9)	
O9	1.0394 (2)	0.62774 (12)	0.08849 (14)	0.0345 (7)	
O10	0.8182 (2)	0.54838 (13)	0.19471 (16)	0.0360 (7)	
O11	1.2344 (2)	0.50741 (15)	0.18357 (16)	0.0408 (8)	
O12	0.2536 (2)	0.69344 (13)	0.69138 (17)	0.0369 (7)	
O13	0.3899 (3)	0.75663 (14)	0.51156 (15)	0.0439 (8)	
O14	0.6441 (2)	0.77508 (14)	0.63612 (18)	0.0406 (8)	
O15	0.23144 (19)	0.82740 (12)	0.66258 (15)	0.0322 (7)	
O16	0.6403 (2)	0.87491 (14)	0.74621 (18)	0.0433 (8)	
O17	0.4574 (3)	0.90584 (15)	0.56021 (16)	0.0492 (9)	
O18	0.3902 (2)	0.95585 (13)	0.79409 (16)	0.0361 (7)	
O19	0.2442 (2)	0.77232 (14)	0.81832 (17)	0.0412 (8)	
O20	0.4848 (3)	0.85964 (15)	0.91846 (16)	0.0458 (8)	
O21	0.4486 (3)	0.66222 (14)	0.90389 (17)	0.0498 (9)	
O22	0.6593 (2)	0.74167 (15)	0.79230 (17)	0.0432 (8)	
C1	1.1235 (3)	0.58298 (15)	0.30176 (18)	0.0203 (7)	
C2	1.0482 (3)	0.53920 (15)	0.41972 (19)	0.0207 (7)	
C3	0.8833 (3)	0.52326 (16)	0.34013 (19)	0.0244 (8)	
C4	1.1568 (3)	0.46479 (15)	0.32219 (18)	0.0221 (8)	
C5	1.0143 (3)	0.40244 (16)	0.3759 (2)	0.0264 (8)	
C6	0.9118 (3)	0.42326 (17)	0.2501 (2)	0.0286 (9)	
C7	1.0891 (3)	0.37462 (17)	0.2386 (2)	0.0304 (9)	
C8	1.0043 (3)	0.46885 (17)	0.1088 (2)	0.0319 (9)	
C9	1.0320 (3)	0.59062 (16)	0.12569 (19)	0.0241 (8)	
C10	0.8953 (3)	0.53866 (17)	0.1970 (2)	0.0282 (8)	
C11	1.1571 (3)	0.51251 (17)	0.18660 (19)	0.0265 (8)	
C12	0.3258 (3)	0.71177 (16)	0.6847 (2)	0.0238 (8)	
C13	0.4142 (3)	0.74812 (16)	0.5685 (2)	0.0254 (8)	
C14	0.5708 (3)	0.76263 (17)	0.6500 (2)	0.0278 (8)	
C15	0.3077 (3)	0.83411 (16)	0.6796 (2)	0.0253 (8)	
C16	0.5653 (3)	0.86347 (17)	0.7305 (2)	0.0292 (9)	
C17	0.4476 (3)	0.88626 (17)	0.6131 (2)	0.0298 (9)	
C18	0.4064 (3)	0.91780 (17)	0.7609 (2)	0.0270 (8)	
C19	0.3214 (3)	0.77160 (17)	0.8105 (2)	0.0280 (8)	
C20	0.4736 (3)	0.82438 (19)	0.8778 (2)	0.0317 (9)	
C21	0.4514 (3)	0.69921 (18)	0.8662 (2)	0.0322 (9)	
C22	0.5825 (3)	0.75258 (19)	0.7934 (2)	0.0323 (9)	
C31	0.8391 (2)	0.65704 (16)	0.36328 (17)	0.0198 (7)	
H31A	0.8187	0.6970	0.3544	0.024*	
H31B	0.7965	0.6316	0.3354	0.024*	
C32	0.8319 (2)	0.64348 (16)	0.44098 (17)	0.0200 (7)	
H32A	0.8540	0.6040	0.4505	0.024*	
H32B	0.8721	0.6700	0.4693	0.024*	
C33	0.7331 (2)	0.64916 (16)	0.46227 (18)	0.0205 (7)	
H33A	0.6943	0.6202	0.4368	0.025*	
H33B	0.7093	0.6874	0.4482	0.025*	
C34	0.7245 (2)	0.64145 (15)	0.54074 (17)	0.0187 (7)	
H34A	0.7609	0.6716	0.5662	0.022*	
H34B	0.7508	0.6039	0.5553	0.022*	
C35	0.6248 (2)	0.64463 (16)	0.56047 (18)	0.0206 (7)	
H35A	0.6009	0.6837	0.5505	0.025*	
H35B	0.5873	0.6176	0.5309	0.025*	
C36	0.6130 (2)	0.63060 (15)	0.63721 (18)	0.0192 (7)	
H36A	0.6541	0.6556	0.6669	0.023*	
H36B	0.6320	0.5905	0.6464	0.023*	
C41	1.0235 (2)	0.69543 (15)	0.39386 (17)	0.0178 (7)	
C42	0.9948 (3)	0.75128 (16)	0.4016 (2)	0.0293 (9)	
H42	0.9420	0.7649	0.3750	0.035*	
C43	1.0428 (3)	0.78771 (18)	0.4483 (2)	0.0368 (11)	
H43	1.0233	0.8263	0.4525	0.044*	
C44	1.1183 (3)	0.76827 (17)	0.4884 (2)	0.0305 (9)	
H44	1.1500	0.7930	0.5211	0.037*	
C45	1.1476 (3)	0.71272 (17)	0.4806 (2)	0.0315 (9)	
H45	1.2004	0.6992	0.5074	0.038*	
C46	1.1000 (3)	0.67627 (15)	0.4335 (2)	0.0228 (8)	
H46	1.1204	0.6379	0.4286	0.027*	
C51	0.9510 (2)	0.68938 (14)	0.25241 (17)	0.0173 (7)	
C52	1.0284 (2)	0.71912 (15)	0.23321 (18)	0.0188 (7)	
H52	1.0830	0.7191	0.2631	0.023*	
C53	1.0260 (3)	0.74909 (16)	0.17011 (19)	0.0230 (8)	
H53	1.0791	0.7691	0.1571	0.028*	
C54	0.9466 (3)	0.74960 (16)	0.12667 (19)	0.0224 (8)	
H54	0.9450	0.7699	0.0837	0.027*	
C55	0.8700 (3)	0.72062 (17)	0.1459 (2)	0.0264 (8)	
H55	0.8153	0.7214	0.1160	0.032*	
C56	0.8712 (3)	0.69031 (16)	0.20788 (19)	0.0227 (7)	
H56	0.8177	0.6702	0.2201	0.027*	
C61	0.4299 (3)	0.59277 (15)	0.59983 (18)	0.0195 (7)	
C62	0.4556 (3)	0.53561 (17)	0.5971 (2)	0.0305 (9)	
H62	0.5057	0.5221	0.6268	0.037*	
C63	0.4089 (4)	0.49828 (18)	0.5516 (2)	0.0394 (11)	
H63	0.4276	0.4594	0.5500	0.047*	
C64	0.3367 (4)	0.51669 (19)	0.5091 (2)	0.0433 (12)	
H64	0.3044	0.4907	0.4784	0.052*	
C65	0.3106 (4)	0.5728 (2)	0.5107 (3)	0.0520 (15)	
H65	0.2606	0.5859	0.4806	0.062*	
C66	0.3571 (3)	0.61109 (18)	0.5565 (2)	0.0351 (10)	
H66	0.3382	0.6500	0.5575	0.042*	
C71	0.4879 (3)	0.59843 (15)	0.74115 (18)	0.0205 (7)	
C72	0.5653 (3)	0.58703 (18)	0.78570 (19)	0.0272 (8)	
H72	0.6237	0.6023	0.7758	0.033*	
C73	0.5570 (3)	0.55321 (19)	0.8449 (2)	0.0353 (10)	
H73	0.6099	0.5454	0.8750	0.042*	
C74	0.4733 (3)	0.53110 (18)	0.8601 (2)	0.0370 (11)	
H74	0.4681	0.5083	0.9006	0.044*	
C75	0.3964 (3)	0.54226 (19)	0.8160 (2)	0.0366 (10)	
H75	0.3384	0.5268	0.8263	0.044*	
C76	0.4031 (3)	0.57533 (18)	0.7575 (2)	0.0290 (9)	
H76	0.3496	0.5827	0.7278	0.035*	
C81	0.239 (2)	0.5739 (10)	0.972 (2)	0.118 (7)	0.5
H81A	0.2892	0.6013	0.9653	0.176*	0.5
H81B	0.1992	0.5885	1.0066	0.176*	0.5
H81C	0.2034	0.5687	0.9270	0.176*	0.5
C82	0.2797 (11)	0.5176 (7)	0.9954 (10)	0.090 (4)	0.5
H82A	0.3293	0.5065	0.9650	0.108*	0.5
H82B	0.3070	0.5214	1.0438	0.108*	0.5
C83	0.2068 (10)	0.4723 (5)	0.9928 (8)	0.080 (3)	0.5
H83A	0.1752	0.4716	0.9455	0.096*	0.5
H83B	0.1604	0.4819	1.0265	0.096*	0.5
C84	0.2456 (14)	0.4140 (6)	1.0095 (12)	0.070 (4)	0.5
H84A	0.2467	0.4148	1.0611	0.084*	0.5
H84B	0.1916	0.3899	0.9954	0.084*	0.5
C85	0.3221 (10)	0.3725 (6)	0.9994 (11)	0.084 (4)	0.5
H85A	0.3805	0.3878	1.0212	0.101*	0.5
H85B	0.3297	0.3675	0.9488	0.101*	0.5
C86	0.3012 (11)	0.3156 (5)	1.0314 (7)	0.071 (3)	0.5
H86A	0.3370	0.2856	1.0099	0.106*	0.5
H86B	0.2353	0.3073	1.0236	0.106*	0.5
H86C	0.3178	0.3169	1.0817	0.106*	0.5
C81X	0.2458 (16)	0.5839 (7)	0.9660 (14)	0.070 (5)	0.5
H81D	0.2084	0.6148	0.9839	0.105*	0.5
H81E	0.2325	0.5802	0.9155	0.105*	0.5
H81F	0.3113	0.5926	0.9756	0.105*	0.5
C82X	0.2231 (11)	0.5287 (5)	1.0012 (7)	0.079 (4)	0.5
H82C	0.2327	0.5342	1.0523	0.094*	0.5
H82D	0.1568	0.5206	0.9908	0.094*	0.5
C83X	0.2770 (6)	0.4768 (4)	0.9814 (4)	0.0384 (19)	0.5
H83C	0.3430	0.4814	0.9963	0.046*	0.5
H83D	0.2712	0.4709	0.9302	0.046*	0.5
C84X	0.2368 (13)	0.4269 (4)	1.0182 (8)	0.066 (4)	0.5
H84C	0.2507	0.4307	1.0692	0.079*	0.5
H84D	0.1689	0.4268	1.0093	0.079*	0.5
C85X	0.2750 (12)	0.3718 (5)	0.9938 (7)	0.076 (4)	0.5
H85C	0.3429	0.3718	1.0025	0.091*	0.5
H85D	0.2608	0.3677	0.9429	0.091*	0.5
C86X	0.2345 (13)	0.3222 (7)	1.0313 (9)	0.091 (5)	0.5
H86D	0.2399	0.2873	1.0035	0.137*	0.5
H86E	0.1693	0.3298	1.0380	0.137*	0.5
H86F	0.2679	0.3171	1.0769	0.137*	0.5

### Atomic displacement parameters (Å^2^)

**Table:**

	*U*^11^	*U*^22^	*U*^33^	*U*^12^	*U*^13^	*U*^23^
Ru1	0.01666 (13)	0.01424 (12)	0.01453 (12)	−0.00087 (10)	0.00393 (10)	−0.00007 (10)
Ru2	0.02584 (15)	0.01404 (13)	0.01748 (13)	−0.00170 (11)	0.00262 (11)	−0.00060 (10)
Ru3	0.02418 (15)	0.01772 (14)	0.01422 (12)	−0.00099 (11)	0.00340 (11)	−0.00010 (10)
Ru4	0.01601 (13)	0.01746 (13)	0.01674 (12)	0.00162 (10)	0.00289 (10)	−0.00148 (10)
Ru5	0.01922 (14)	0.01841 (14)	0.02283 (14)	0.00088 (11)	0.00067 (11)	−0.00223 (11)
Ru6	0.02241 (15)	0.02536 (15)	0.01751 (13)	0.00297 (12)	0.00093 (11)	−0.00131 (11)
P1	0.0144 (4)	0.0167 (4)	0.0145 (4)	0.0015 (3)	0.0025 (3)	0.0008 (3)
P2	0.0139 (4)	0.0185 (4)	0.0141 (4)	0.0029 (3)	0.0019 (3)	−0.0010 (3)
O1	0.0216 (14)	0.0248 (14)	0.0404 (16)	−0.0032 (11)	0.0120 (12)	−0.0031 (12)
O2	0.0314 (16)	0.0342 (16)	0.0212 (13)	−0.0039 (12)	0.0010 (12)	0.0042 (11)
O3	0.0275 (16)	0.0411 (18)	0.0425 (17)	−0.0113 (13)	0.0120 (14)	−0.0024 (14)
O4	0.0256 (15)	0.0254 (14)	0.0358 (15)	0.0038 (11)	−0.0036 (12)	0.0049 (12)
O5	0.054 (2)	0.0303 (16)	0.0262 (15)	0.0020 (14)	0.0114 (14)	0.0054 (12)
O6	0.0350 (18)	0.0390 (18)	0.0484 (19)	−0.0153 (14)	−0.0042 (15)	0.0028 (15)
O7	0.060 (2)	0.0301 (17)	0.049 (2)	0.0015 (16)	0.0112 (17)	−0.0189 (15)
O8	0.074 (3)	0.0392 (19)	0.0406 (18)	−0.0099 (18)	0.0030 (18)	−0.0195 (15)
O9	0.049 (2)	0.0286 (15)	0.0264 (14)	0.0004 (14)	0.0090 (14)	0.0064 (12)
O10	0.0250 (16)	0.0443 (18)	0.0386 (17)	0.0007 (13)	0.0007 (13)	0.0064 (14)
O11	0.0322 (18)	0.060 (2)	0.0309 (16)	0.0070 (16)	0.0089 (14)	−0.0048 (15)
O12	0.0198 (15)	0.0328 (16)	0.059 (2)	−0.0018 (12)	0.0098 (14)	−0.0007 (14)
O13	0.066 (2)	0.0432 (19)	0.0221 (15)	−0.0032 (17)	−0.0021 (15)	0.0055 (13)
O14	0.0258 (16)	0.0427 (18)	0.055 (2)	−0.0092 (14)	0.0167 (15)	−0.0095 (15)
O15	0.0211 (14)	0.0338 (16)	0.0414 (17)	0.0017 (12)	−0.0020 (13)	−0.0043 (13)
O16	0.0240 (16)	0.0417 (18)	0.063 (2)	−0.0034 (14)	−0.0036 (15)	−0.0047 (16)
O17	0.071 (3)	0.046 (2)	0.0303 (17)	−0.0151 (18)	0.0022 (17)	0.0059 (15)
O18	0.0316 (17)	0.0302 (16)	0.0464 (18)	0.0020 (13)	0.0010 (14)	−0.0147 (14)
O19	0.0272 (17)	0.052 (2)	0.0455 (19)	0.0074 (15)	0.0094 (14)	0.0025 (15)
O20	0.059 (2)	0.047 (2)	0.0305 (16)	−0.0028 (17)	−0.0030 (16)	−0.0131 (15)
O21	0.082 (3)	0.0366 (18)	0.0331 (17)	0.0175 (18)	0.0206 (18)	0.0088 (14)
O22	0.0216 (16)	0.060 (2)	0.0481 (19)	0.0081 (15)	0.0000 (14)	0.0062 (16)
C1	0.0236 (19)	0.0161 (16)	0.0219 (17)	0.0051 (14)	0.0086 (14)	0.0005 (13)
C2	0.0226 (18)	0.0166 (16)	0.0233 (17)	−0.0038 (14)	0.0043 (15)	−0.0003 (13)
C3	0.025 (2)	0.0247 (19)	0.0236 (18)	−0.0047 (16)	0.0045 (15)	−0.0038 (15)
C4	0.033 (2)	0.0137 (16)	0.0198 (17)	0.0041 (15)	0.0036 (15)	0.0025 (13)
C5	0.036 (2)	0.0178 (18)	0.0256 (19)	−0.0004 (16)	0.0028 (17)	−0.0005 (15)
C6	0.034 (2)	0.026 (2)	0.0257 (19)	−0.0055 (17)	0.0029 (17)	0.0018 (16)
C7	0.040 (2)	0.022 (2)	0.029 (2)	−0.0044 (17)	0.0042 (18)	−0.0033 (16)
C8	0.045 (3)	0.024 (2)	0.027 (2)	−0.0050 (18)	0.0054 (19)	−0.0004 (16)
C9	0.028 (2)	0.0231 (19)	0.0209 (17)	−0.0001 (15)	0.0024 (15)	−0.0022 (15)
C10	0.029 (2)	0.030 (2)	0.0259 (19)	−0.0018 (17)	0.0042 (17)	0.0068 (16)
C11	0.033 (2)	0.031 (2)	0.0168 (17)	0.0048 (17)	0.0058 (16)	−0.0040 (15)
C12	0.0221 (19)	0.0218 (18)	0.0279 (19)	0.0035 (15)	0.0040 (16)	−0.0027 (15)
C13	0.028 (2)	0.0223 (18)	0.0261 (19)	0.0011 (15)	0.0039 (16)	−0.0007 (15)
C14	0.023 (2)	0.030 (2)	0.031 (2)	−0.0002 (16)	0.0042 (16)	−0.0046 (16)
C15	0.026 (2)	0.0242 (19)	0.0261 (19)	−0.0027 (15)	0.0028 (16)	−0.0002 (15)
C16	0.026 (2)	0.027 (2)	0.035 (2)	−0.0002 (16)	−0.0009 (17)	−0.0044 (17)
C17	0.034 (2)	0.024 (2)	0.031 (2)	−0.0045 (17)	−0.0021 (18)	−0.0025 (16)
C18	0.0218 (19)	0.027 (2)	0.032 (2)	−0.0015 (16)	−0.0003 (16)	−0.0021 (17)
C19	0.029 (2)	0.030 (2)	0.0258 (19)	0.0023 (17)	0.0006 (17)	0.0018 (16)
C20	0.033 (2)	0.037 (2)	0.0249 (19)	−0.0011 (18)	−0.0023 (17)	−0.0025 (17)
C21	0.042 (3)	0.032 (2)	0.0234 (19)	0.0124 (19)	0.0073 (18)	−0.0016 (17)
C22	0.030 (2)	0.036 (2)	0.031 (2)	0.0017 (18)	−0.0011 (18)	0.0030 (17)
C31	0.0165 (17)	0.0271 (19)	0.0161 (16)	0.0026 (14)	0.0035 (13)	0.0013 (13)
C32	0.0168 (17)	0.0274 (19)	0.0163 (15)	0.0020 (14)	0.0053 (13)	−0.0005 (14)
C33	0.0193 (17)	0.0253 (18)	0.0175 (16)	0.0034 (14)	0.0058 (14)	0.0026 (14)
C34	0.0175 (17)	0.0211 (17)	0.0181 (16)	0.0013 (13)	0.0059 (13)	0.0008 (13)
C35	0.0186 (17)	0.0252 (18)	0.0186 (16)	0.0027 (14)	0.0055 (14)	−0.0007 (14)
C36	0.0168 (17)	0.0226 (18)	0.0187 (16)	0.0024 (13)	0.0042 (13)	−0.0012 (13)
C41	0.0194 (17)	0.0185 (16)	0.0158 (15)	−0.0005 (13)	0.0031 (13)	0.0011 (13)
C42	0.040 (2)	0.0215 (19)	0.0254 (19)	0.0076 (17)	−0.0045 (18)	−0.0027 (15)
C43	0.054 (3)	0.020 (2)	0.035 (2)	0.0061 (19)	−0.006 (2)	−0.0058 (17)
C44	0.038 (2)	0.023 (2)	0.030 (2)	−0.0081 (17)	0.0004 (18)	−0.0085 (16)
C45	0.024 (2)	0.029 (2)	0.040 (2)	−0.0003 (16)	−0.0081 (18)	−0.0049 (17)
C46	0.0200 (18)	0.0179 (17)	0.0302 (19)	0.0019 (14)	−0.0001 (15)	−0.0024 (14)
C51	0.0190 (17)	0.0146 (15)	0.0186 (15)	0.0039 (13)	0.0047 (13)	−0.0007 (13)
C52	0.0175 (17)	0.0207 (17)	0.0184 (16)	−0.0010 (13)	0.0016 (13)	0.0015 (13)
C53	0.0207 (18)	0.0220 (18)	0.0270 (18)	0.0010 (14)	0.0056 (15)	0.0029 (15)
C54	0.0242 (19)	0.0231 (18)	0.0205 (17)	0.0049 (15)	0.0051 (15)	0.0073 (14)
C55	0.0211 (19)	0.033 (2)	0.0246 (18)	0.0023 (16)	−0.0009 (15)	0.0076 (16)
C56	0.0167 (17)	0.0284 (19)	0.0231 (17)	0.0008 (15)	0.0019 (14)	0.0046 (15)
C61	0.0219 (18)	0.0198 (17)	0.0172 (16)	−0.0006 (14)	0.0040 (14)	−0.0013 (13)
C62	0.037 (2)	0.0216 (19)	0.032 (2)	0.0038 (17)	−0.0040 (18)	−0.0050 (16)
C63	0.051 (3)	0.022 (2)	0.044 (3)	−0.0002 (19)	0.002 (2)	−0.0108 (18)
C64	0.054 (3)	0.032 (2)	0.043 (3)	−0.013 (2)	−0.012 (2)	−0.011 (2)
C65	0.054 (3)	0.037 (3)	0.060 (3)	−0.006 (2)	−0.034 (3)	−0.003 (2)
C66	0.035 (2)	0.022 (2)	0.046 (2)	0.0008 (17)	−0.015 (2)	−0.0016 (18)
C71	0.0217 (18)	0.0206 (17)	0.0194 (16)	0.0045 (14)	0.0044 (14)	−0.0022 (13)
C72	0.0234 (19)	0.036 (2)	0.0227 (18)	0.0101 (17)	0.0040 (15)	0.0029 (16)
C73	0.039 (2)	0.045 (3)	0.0228 (19)	0.020 (2)	0.0064 (18)	0.0086 (18)
C74	0.054 (3)	0.030 (2)	0.029 (2)	0.015 (2)	0.018 (2)	0.0111 (17)
C75	0.041 (3)	0.037 (2)	0.033 (2)	−0.004 (2)	0.015 (2)	0.0049 (19)
C76	0.027 (2)	0.034 (2)	0.0267 (19)	−0.0002 (17)	0.0054 (16)	0.0040 (16)
C81	0.120 (11)	0.107 (8)	0.125 (11)	−0.002 (8)	0.005 (9)	0.011 (8)
C82	0.087 (6)	0.094 (5)	0.089 (6)	−0.004 (4)	0.008 (4)	0.008 (4)
C83	0.086 (5)	0.083 (4)	0.071 (5)	0.001 (4)	−0.003 (4)	−0.002 (4)
C84	0.077 (6)	0.069 (5)	0.063 (6)	0.003 (4)	0.000 (4)	0.000 (4)
C85	0.089 (6)	0.082 (5)	0.082 (6)	−0.007 (4)	0.008 (5)	−0.007 (4)
C86	0.094 (8)	0.059 (5)	0.056 (6)	0.009 (6)	−0.012 (6)	−0.003 (5)
C81X	0.074 (8)	0.073 (6)	0.065 (7)	0.023 (6)	0.024 (6)	0.014 (6)
C82X	0.083 (9)	0.091 (7)	0.066 (7)	0.045 (7)	0.036 (7)	0.047 (7)
C83X	0.038 (5)	0.053 (5)	0.023 (4)	−0.016 (4)	−0.008 (4)	−0.005 (4)
C84X	0.101 (10)	0.059 (6)	0.035 (6)	−0.001 (6)	−0.016 (6)	−0.015 (5)
C85X	0.123 (13)	0.063 (6)	0.044 (6)	0.007 (8)	0.017 (8)	−0.018 (5)
C86X	0.112 (9)	0.083 (7)	0.079 (8)	−0.023 (7)	0.013 (7)	−0.004 (6)

### Geometric parameters (Å, º)

**Table:**

Ru1—C2	1.888 (4)	C43—C44	1.377 (6)
Ru1—C1	1.927 (4)	C43—H43	0.9500
Ru1—C3	1.951 (4)	C44—C45	1.377 (6)
Ru1—P1	2.3506 (9)	C44—H44	0.9500
Ru1—Ru2	2.8431 (4)	C45—C46	1.393 (5)
Ru1—Ru3	2.8644 (4)	C45—H45	0.9500
Ru2—C4	1.926 (4)	C46—H46	0.9500
Ru2—C7	1.926 (4)	C51—C52	1.392 (5)
Ru2—C5	1.927 (4)	C51—C56	1.396 (5)
Ru2—C6	1.948 (4)	C52—C53	1.397 (5)
Ru2—Ru3	2.8378 (4)	C52—H52	0.9500
Ru3—C8	1.913 (4)	C53—C54	1.379 (5)
Ru3—C9	1.928 (4)	C53—H53	0.9500
Ru3—C10	1.941 (4)	C54—C55	1.374 (5)
Ru3—C11	1.948 (4)	C54—H54	0.9500
Ru4—C13	1.885 (4)	C55—C56	1.384 (5)
Ru4—C14	1.927 (4)	C55—H55	0.9500
Ru4—C12	1.928 (4)	C56—H56	0.9500
Ru4—P2	2.3439 (9)	C61—C66	1.372 (5)
Ru4—Ru5	2.8454 (4)	C61—C62	1.389 (5)
Ru4—Ru6	2.8564 (4)	C62—C63	1.381 (6)
Ru5—C17	1.926 (4)	C62—H62	0.9500
Ru5—C18	1.929 (4)	C63—C64	1.356 (7)
Ru5—C15	1.933 (4)	C63—H63	0.9500
Ru5—C16	1.942 (4)	C64—C65	1.367 (7)
Ru5—Ru6	2.8490 (4)	C64—H64	0.9500
Ru6—C20	1.908 (4)	C65—C66	1.396 (6)
Ru6—C22	1.933 (5)	C65—H65	0.9500
Ru6—C21	1.926 (4)	C66—H66	0.9500
Ru6—C19	1.936 (4)	C71—C72	1.395 (5)
P1—C51	1.829 (3)	C71—C76	1.399 (5)
P1—C31	1.834 (4)	C72—C73	1.396 (5)
P1—C41	1.836 (3)	C72—H72	0.9500
P2—C71	1.822 (4)	C73—C74	1.370 (7)
P2—C61	1.830 (4)	C73—H73	0.9500
P2—C36	1.834 (4)	C74—C75	1.383 (7)
O1—C1	1.141 (4)	C74—H74	0.9500
O2—C2	1.148 (4)	C75—C76	1.370 (6)
O3—C3	1.131 (5)	C75—H75	0.9500
O4—C4	1.148 (5)	C76—H76	0.9500
O5—C5	1.133 (5)	C81—C82	1.499 (5)
O6—C6	1.140 (5)	C81—H81A	0.9800
O7—C7	1.139 (5)	C81—H81B	0.9800
O8—C8	1.138 (5)	C81—H81C	0.9800
O9—C9	1.133 (5)	C82—C83	1.496 (5)
O10—C10	1.142 (5)	C82—H82A	0.9900
O11—C11	1.135 (5)	C82—H82B	0.9900
O12—C12	1.148 (5)	C83—C84	1.502 (5)
O13—C13	1.144 (5)	C83—H83A	0.9900
O14—C14	1.151 (5)	C83—H83B	0.9900
O15—C15	1.147 (5)	C84—C85	1.497 (5)
O16—C16	1.143 (5)	C84—H84A	0.9900
O17—C17	1.131 (5)	C84—H84B	0.9900
O18—C18	1.128 (5)	C85—C86	1.504 (5)
O19—C19	1.141 (5)	C85—H85A	0.9900
O20—C20	1.139 (5)	C85—H85B	0.9900
O21—C21	1.130 (5)	C86—H86A	0.9800
O22—C22	1.146 (5)	C86—H86B	0.9800
C31—C32	1.534 (5)	C86—H86C	0.9800
C31—H31A	0.9900	C81X—C82X	1.503 (5)
C31—H31B	0.9900	C81X—H81D	0.9800
C32—C33	1.524 (5)	C81X—H81E	0.9800
C32—H32A	0.9900	C81X—H81F	0.9800
C32—H32B	0.9900	C82X—C83X	1.506 (5)
C33—C34	1.529 (5)	C82X—H82C	0.9900
C33—H33A	0.9900	C82X—H82D	0.9900
C33—H33B	0.9900	C83X—C84X	1.501 (5)
C34—C35	1.523 (5)	C83X—H83C	0.9900
C34—H34A	0.9900	C83X—H83D	0.9900
C34—H34B	0.9900	C84X—C85X	1.489 (5)
C35—C36	1.529 (5)	C84X—H84C	0.9900
C35—H35A	0.9900	C84X—H84D	0.9900
C35—H35B	0.9900	C85X—C86X	1.505 (5)
C36—H36A	0.9900	C85X—H85C	0.9900
C36—H36B	0.9900	C85X—H85D	0.9900
C41—C42	1.381 (5)	C86X—H86D	0.9800
C41—C46	1.381 (5)	C86X—H86E	0.9800
C42—C43	1.390 (6)	C86X—H86F	0.9800
C42—H42	0.9500		
			
C2—Ru1—C1	93.19 (15)	C34—C35—H35B	108.9
C2—Ru1—C3	92.90 (16)	H35A—C35—H35B	107.7
C1—Ru1—C3	173.85 (15)	C35—C36—P2	112.5 (2)
C2—Ru1—P1	100.79 (11)	C35—C36—H36A	109.1
C1—Ru1—P1	87.47 (10)	P2—C36—H36A	109.1
C3—Ru1—P1	92.18 (12)	C35—C36—H36B	109.1
C2—Ru1—Ru2	92.04 (11)	P2—C36—H36B	109.1
C1—Ru1—Ru2	97.12 (10)	H36A—C36—H36B	107.8
C3—Ru1—Ru2	81.85 (11)	C42—C41—C46	119.0 (3)
P1—Ru1—Ru2	166.14 (2)	C42—C41—P1	118.8 (3)
C2—Ru1—Ru3	145.87 (11)	C46—C41—P1	122.2 (3)
C1—Ru1—Ru3	73.77 (11)	C41—C42—C43	120.4 (4)
C3—Ru1—Ru3	100.62 (11)	C41—C42—H42	119.8
P1—Ru1—Ru3	109.75 (2)	C43—C42—H42	119.8
Ru2—Ru1—Ru3	59.630 (9)	C44—C43—C42	120.4 (4)
C4—Ru2—C7	92.79 (17)	C44—C43—H43	119.8
C4—Ru2—C5	92.47 (16)	C42—C43—H43	119.8
C7—Ru2—C5	101.53 (17)	C45—C44—C43	119.4 (4)
C4—Ru2—C6	170.51 (16)	C45—C44—H44	120.3
C7—Ru2—C6	94.20 (18)	C43—C44—H44	120.3
C5—Ru2—C6	92.43 (17)	C44—C45—C46	120.2 (4)
C4—Ru2—Ru3	91.68 (10)	C44—C45—H45	119.9
C7—Ru2—Ru3	101.66 (12)	C46—C45—H45	119.9
C5—Ru2—Ru3	156.20 (12)	C41—C46—C45	120.5 (3)
C6—Ru2—Ru3	80.62 (12)	C41—C46—H46	119.7
C4—Ru2—Ru1	75.64 (11)	C45—C46—H46	119.7
C7—Ru2—Ru1	157.76 (13)	C52—C51—C56	119.0 (3)
C5—Ru2—Ru1	97.94 (11)	C52—C51—P1	120.0 (3)
C6—Ru2—Ru1	95.63 (12)	C56—C51—P1	121.0 (3)
Ru3—Ru2—Ru1	60.559 (10)	C51—C52—C53	120.3 (3)
C8—Ru3—C9	97.59 (16)	C51—C52—H52	119.9
C8—Ru3—C10	97.34 (19)	C53—C52—H52	119.9
C9—Ru3—C10	91.23 (17)	C54—C53—C52	120.1 (3)
C8—Ru3—C11	92.51 (18)	C54—C53—H53	119.9
C9—Ru3—C11	91.69 (17)	C52—C53—H53	119.9
C10—Ru3—C11	169.27 (16)	C55—C54—C53	119.7 (3)
C8—Ru3—Ru2	93.38 (12)	C55—C54—H54	120.2
C9—Ru3—Ru2	167.57 (11)	C53—C54—H54	120.2
C10—Ru3—Ru2	93.16 (11)	C54—C55—C56	121.1 (4)
C11—Ru3—Ru2	81.97 (12)	C54—C55—H55	119.4
C8—Ru3—Ru1	148.89 (13)	C56—C55—H55	119.4
C9—Ru3—Ru1	111.03 (11)	C55—C56—C51	119.9 (3)
C10—Ru3—Ru1	70.94 (12)	C55—C56—H56	120.1
C11—Ru3—Ru1	98.38 (11)	C51—C56—H56	120.1
Ru2—Ru3—Ru1	59.811 (9)	C66—C61—C62	118.4 (3)
C13—Ru4—C14	90.16 (18)	C66—C61—P2	122.9 (3)
C13—Ru4—C12	93.49 (17)	C62—C61—P2	118.7 (3)
C14—Ru4—C12	175.82 (16)	C63—C62—C61	120.6 (4)
C13—Ru4—P2	100.25 (12)	C63—C62—H62	119.7
C14—Ru4—P2	91.98 (12)	C61—C62—H62	119.7
C12—Ru4—P2	89.38 (11)	C64—C63—C62	120.6 (4)
C13—Ru4—Ru5	95.90 (12)	C64—C63—H63	119.7
C14—Ru4—Ru5	78.78 (12)	C62—C63—H63	119.7
C12—Ru4—Ru5	98.80 (11)	C63—C64—C65	119.8 (4)
P2—Ru4—Ru5	161.41 (2)	C63—C64—H64	120.1
C13—Ru4—Ru6	153.66 (12)	C65—C64—H64	120.1
C14—Ru4—Ru6	94.70 (12)	C64—C65—C66	120.4 (4)
C12—Ru4—Ru6	81.13 (11)	C64—C65—H65	119.8
P2—Ru4—Ru6	105.42 (2)	C66—C65—H65	119.8
Ru5—Ru4—Ru6	59.956 (10)	C61—C66—C65	120.3 (4)
C17—Ru5—C18	103.41 (17)	C61—C66—H66	119.9
C17—Ru5—C15	91.02 (17)	C65—C66—H66	119.9
C18—Ru5—C15	94.36 (17)	C72—C71—C76	118.4 (4)
C17—Ru5—C16	92.36 (18)	C72—C71—P2	122.5 (3)
C18—Ru5—C16	89.77 (17)	C76—C71—P2	119.1 (3)
C15—Ru5—C16	173.92 (17)	C73—C72—C71	119.9 (4)
C17—Ru5—Ru4	97.59 (12)	C73—C72—H72	120.0
C18—Ru5—Ru4	157.80 (12)	C71—C72—H72	120.0
C15—Ru5—Ru4	78.01 (12)	C74—C73—C72	120.7 (4)
C16—Ru5—Ru4	96.53 (12)	C74—C73—H73	119.6
C17—Ru5—Ru6	155.84 (13)	C72—C73—H73	119.6
C18—Ru5—Ru6	99.99 (12)	C73—C74—C75	119.5 (4)
C15—Ru5—Ru6	93.16 (11)	C73—C74—H74	120.3
C16—Ru5—Ru6	81.72 (13)	C75—C74—H74	120.3
Ru4—Ru5—Ru6	60.213 (10)	C76—C75—C74	120.7 (4)
C20—Ru6—C22	96.98 (19)	C76—C75—H75	119.6
C20—Ru6—C21	100.63 (18)	C74—C75—H75	119.6
C22—Ru6—C21	90.24 (19)	C75—C76—C71	120.7 (4)
C20—Ru6—C19	92.56 (18)	C75—C76—H76	119.6
C22—Ru6—C19	170.42 (18)	C71—C76—H76	119.6
C21—Ru6—C19	88.92 (18)	C82—C81—H81A	109.5
C20—Ru6—Ru5	89.47 (13)	C82—C81—H81B	109.5
C22—Ru6—Ru5	93.73 (13)	H81A—C81—H81B	109.5
C21—Ru6—Ru5	168.62 (13)	C82—C81—H81C	109.5
C19—Ru6—Ru5	85.39 (12)	H81A—C81—H81C	109.5
C20—Ru6—Ru4	147.96 (13)	H81B—C81—H81C	109.5
C22—Ru6—Ru4	77.65 (12)	C83—C82—C81	110.4 (17)
C21—Ru6—Ru4	110.86 (12)	C83—C82—H82A	109.6
C19—Ru6—Ru4	93.74 (12)	C81—C82—H82A	109.6
Ru5—Ru6—Ru4	59.831 (10)	C83—C82—H82B	109.6
C51—P1—C31	102.71 (16)	C81—C82—H82B	109.6
C51—P1—C41	102.18 (16)	H82A—C82—H82B	108.1
C31—P1—C41	102.02 (16)	C82—C83—C84	112.4 (15)
C51—P1—Ru1	116.05 (11)	C82—C83—H83A	109.1
C31—P1—Ru1	115.00 (12)	C84—C83—H83A	109.1
C41—P1—Ru1	116.74 (11)	C82—C83—H83B	109.1
C71—P2—C61	99.68 (16)	C84—C83—H83B	109.1
C71—P2—C36	104.11 (16)	H83A—C83—H83B	107.9
C61—P2—C36	102.27 (16)	C85—C84—C83	146.3 (16)
C71—P2—Ru4	117.77 (12)	C85—C84—H84A	100.3
C61—P2—Ru4	117.29 (12)	C83—C84—H84A	100.3
C36—P2—Ru4	113.47 (12)	C85—C84—H84B	100.3
O1—C1—Ru1	171.7 (3)	C83—C84—H84B	100.3
O2—C2—Ru1	174.1 (3)	H84A—C84—H84B	104.2
O3—C3—Ru1	175.7 (3)	C84—C85—C86	110.4 (11)
O4—C4—Ru2	174.4 (3)	C84—C85—H85A	109.6
O5—C5—Ru2	178.7 (4)	C86—C85—H85A	109.6
O6—C6—Ru2	176.2 (4)	C84—C85—H85B	109.6
O7—C7—Ru2	174.2 (4)	C86—C85—H85B	109.6
O8—C8—Ru3	178.7 (4)	H85A—C85—H85B	108.1
O9—C9—Ru3	174.9 (3)	C85—C86—H86A	109.5
O10—C10—Ru3	169.7 (4)	C85—C86—H86B	109.5
O11—C11—Ru3	174.8 (3)	H86A—C86—H86B	109.5
O12—C12—Ru4	172.9 (3)	C85—C86—H86C	109.5
O13—C13—Ru4	176.6 (4)	H86A—C86—H86C	109.5
O14—C14—Ru4	173.4 (4)	H86B—C86—H86C	109.5
O15—C15—Ru5	174.1 (3)	C82X—C81X—H81D	109.5
O16—C16—Ru5	173.7 (4)	C82X—C81X—H81E	109.5
O17—C17—Ru5	178.1 (4)	H81D—C81X—H81E	109.5
O18—C18—Ru5	179.4 (4)	C82X—C81X—H81F	109.5
O19—C19—Ru6	173.2 (4)	H81D—C81X—H81F	109.5
O20—C20—Ru6	178.9 (4)	H81E—C81X—H81F	109.5
O21—C21—Ru6	175.2 (4)	C83X—C82X—C81X	116.5 (12)
O22—C22—Ru6	171.9 (4)	C83X—C82X—H82C	108.2
C32—C31—P1	113.5 (2)	C81X—C82X—H82C	108.2
C32—C31—H31A	108.9	C83X—C82X—H82D	108.2
P1—C31—H31A	108.9	C81X—C82X—H82D	108.2
C32—C31—H31B	108.9	H82C—C82X—H82D	107.3
P1—C31—H31B	108.9	C82X—C83X—C84X	106.5 (9)
H31A—C31—H31B	107.7	C82X—C83X—H83C	110.4
C33—C32—C31	111.5 (3)	C84X—C83X—H83C	110.4
C33—C32—H32A	109.3	C82X—C83X—H83D	110.4
C31—C32—H32A	109.3	C84X—C83X—H83D	110.4
C33—C32—H32B	109.3	H83C—C83X—H83D	108.6
C31—C32—H32B	109.3	C85X—C84X—C83X	111.0 (10)
H32A—C32—H32B	108.0	C85X—C84X—H84C	109.4
C32—C33—C34	113.0 (3)	C83X—C84X—H84C	109.4
C32—C33—H33A	109.0	C85X—C84X—H84D	109.4
C34—C33—H33A	109.0	C83X—C84X—H84D	109.4
C32—C33—H33B	109.0	H84C—C84X—H84D	108.0
C34—C33—H33B	109.0	C84X—C85X—C86X	110.5 (12)
H33A—C33—H33B	107.8	C84X—C85X—H85C	109.6
C33—C34—C35	112.2 (3)	C86X—C85X—H85C	109.6
C33—C34—H34A	109.2	C84X—C85X—H85D	109.6
C35—C34—H34A	109.2	C86X—C85X—H85D	109.6
C33—C34—H34B	109.2	H85C—C85X—H85D	108.1
C35—C34—H34B	109.2	C85X—C86X—H86D	109.5
H34A—C34—H34B	107.9	C85X—C86X—H86E	109.5
C36—C35—C34	113.3 (3)	H86D—C86X—H86E	109.5
C36—C35—H35A	108.9	C85X—C86X—H86F	109.5
C34—C35—H35A	108.9	H86D—C86X—H86F	109.5
C36—C35—H35B	108.9	H86E—C86X—H86F	109.5
			
C51—P1—C31—C32	−161.5 (3)	C52—C51—C56—C55	−0.1 (5)
C41—P1—C31—C32	−55.9 (3)	P1—C51—C56—C55	−178.5 (3)
Ru1—P1—C31—C32	71.5 (3)	C71—P2—C61—C66	127.9 (4)
P1—C31—C32—C33	−177.8 (3)	C36—P2—C61—C66	−125.2 (4)
C31—C32—C33—C34	−174.6 (3)	Ru4—P2—C61—C66	−0.4 (4)
C32—C33—C34—C35	−177.4 (3)	C71—P2—C61—C62	−51.7 (3)
C33—C34—C35—C36	173.7 (3)	C36—P2—C61—C62	55.2 (3)
C34—C35—C36—P2	175.5 (2)	Ru4—P2—C61—C62	−180.0 (3)
C71—P2—C36—C35	160.6 (3)	C66—C61—C62—C63	0.2 (6)
C61—P2—C36—C35	57.2 (3)	P2—C61—C62—C63	179.8 (4)
Ru4—P2—C36—C35	−70.1 (3)	C61—C62—C63—C64	−0.5 (7)
C51—P1—C41—C42	54.3 (3)	C62—C63—C64—C65	0.9 (8)
C31—P1—C41—C42	−51.8 (3)	C63—C64—C65—C66	−0.9 (9)
Ru1—P1—C41—C42	−178.0 (3)	C62—C61—C66—C65	−0.2 (7)
C51—P1—C41—C46	−128.3 (3)	P2—C61—C66—C65	−179.8 (4)
C31—P1—C41—C46	125.7 (3)	C64—C65—C66—C61	0.5 (8)
Ru1—P1—C41—C46	−0.6 (4)	C61—P2—C71—C72	131.1 (3)
C46—C41—C42—C43	0.5 (6)	C36—P2—C71—C72	25.7 (4)
P1—C41—C42—C43	178.0 (3)	Ru4—P2—C71—C72	−100.9 (3)
C41—C42—C43—C44	−1.4 (7)	C61—P2—C71—C76	−46.9 (3)
C42—C43—C44—C45	1.7 (7)	C36—P2—C71—C76	−152.2 (3)
C43—C44—C45—C46	−1.2 (7)	Ru4—P2—C71—C76	81.1 (3)
C42—C41—C46—C45	0.0 (6)	C76—C71—C72—C73	0.1 (6)
P1—C41—C46—C45	−177.5 (3)	P2—C71—C72—C73	−177.9 (3)
C44—C45—C46—C41	0.4 (6)	C71—C72—C73—C74	−0.2 (6)
C31—P1—C51—C52	145.4 (3)	C72—C73—C74—C75	0.3 (7)
C41—P1—C51—C52	39.9 (3)	C73—C74—C75—C76	−0.3 (7)
Ru1—P1—C51—C52	−88.3 (3)	C74—C75—C76—C71	0.2 (7)
C31—P1—C51—C56	−36.3 (3)	C72—C71—C76—C75	−0.1 (6)
C41—P1—C51—C56	−141.8 (3)	P2—C71—C76—C75	178.0 (3)
Ru1—P1—C51—C56	90.1 (3)	C81—C82—C83—C84	174 (2)
C56—C51—C52—C53	−0.4 (5)	C82—C83—C84—C85	−42 (4)
P1—C51—C52—C53	178.0 (3)	C83—C84—C85—C86	−174 (3)
C51—C52—C53—C54	0.4 (5)	C81X—C82X—C83X—C84X	−175.3 (17)
C52—C53—C54—C55	0.1 (6)	C82X—C83X—C84X—C85X	171.2 (13)
C53—C54—C55—C56	−0.7 (6)	C83X—C84X—C85X—C86X	179.7 (14)
C54—C55—C56—C51	0.7 (6)		

### Hydrogen-bond geometry (Å, º)

Hydrogen-bond geometry (Å, °) for (I). *Cg*1 and *Cg*2 the ring centroids of the C41–C46 and C51–C56 rings, respectively.

**Table:**

*D*—H···*A*	*D*—H	H···*A*	*D*···*A*	*D*—H···*A*
C35—H35*B*···O20^i^	0.99	2.60	3.297 (5)	128
C36—H36*B*···O4^ii^	0.99	2.54	3.393 (4)	144
C42—H42···O7^iii^	0.95	2.56	3.401 (5)	148
C52—H52···O19^iv^	0.95	2.52	3.448 (4)	167
C55—H55···O14^i^	0.95	2.54	3.278 (5)	134
C62—H62···O18^v^	0.95	2.59	3.501 (5)	161
C82*X*—H82*D*···O8^vi^	0.99	2.55	3.391 (16)	143
C81*X*—H81*F*···O17^vii^	0.98	2.59	3.48 (2)	150
C82*X*—H82*C*···O11^viii^	0.99	2.59	3.528 (14)	157

Symmetry codes: (i) *x*, −*y*+1/2, *z*−3/2; (ii) −*x*+2, −*y*+1, −*z*+1; (iii) −*x*+2, *y*+1/2, −*z*+1/2; (iv) *x*+1, −*y*+1/2, *z*−3/2; (v) −*x*+1, *y*−1/2, −*z*+3/2; (vi) −*x*+1, −*y*+1, −*z*+1; (vii) *x*, −*y*+1/2, *z*−1/2; (viii) *x*−1, *y*, *z*+1.
